# Rehabilitation in the long-term care insurance domain: a scoping review

**DOI:** 10.1186/s13561-022-00407-6

**Published:** 2022-12-01

**Authors:** Hiroshi Shinohara, Yukio Mikami, Rumi Kuroda, Makoto Asaeda, Takashi Kawasaki, Ken Kouda, Yukihide Nishimura, Hiroyuki Ohkawa, Hiroyasu Uenishi, Toshio Shimokawa, Yasuo Mikami, Fumihiro Tajima, Toshikazu Kubo

**Affiliations:** 1 Graduate School of Health Science, Aomori University of Health and Welfare, 58-1 Mase, 030-8505 Hamadate, Aomori Japan; 2 Department of Rehabilitation Medicine, Hiroshima University Hospital, 1-2-3 Kasumi, Minami-ku, 734-8551 Hiroshima, Japan; 3 School of Nursing, Fukushima Medical University, 1 Hikariga-oka, 960-1295 Fukushima, Japan; 4 Faculty of Wakayama Health Care Sciences, Takarazuka University of Medical and Health Care, 2252, 640-8392 Nakanoshima, Wakayama, Japan; 5 Department of Rehabilitation Medicine, Graduate School of Medical Science, Kyoto Prefectural University of Medicine, 465, Kawaramachi-Hirokoji, Kamigyo-ku, 602-8566 Kyoto, Japan; 6 Department of Rehabilitation Medicine, Iwate Medical University, 1-1-1 Idaidori, Yahaba-cho, Shiwa-gun, Iwate, 028-3694 Iwate, Japan; 7 Department of Health and Nutrition Sciences, Faculty of Health and Nutrition Sciences, Nishikyushu University, 4490-9 Osaki, Kanzaki-machi, 842-8585 Kanzaki, Saga Japan; 8 Clinical Study Support Center, Wakayama Medical University, 811-1 Kimiidera, 641-8509 Wakayama, Japan; 9 Department of Orthopedics, Graduate School of Medical Science, Kyoto Prefectural University of Medicine, 465, Kawaramachi-Hirokoji, Kamigyo-ku, 602-8566 Kyoto, Japan

**Keywords:** Dementia, Fall prevention, Long-term-care insurance, Occupational therapy model, Rehabilitation

## Abstract

**Purpose:**

Since the enactment of the long-term care insurance (LTCI) act in 2000, the number of LTCI users has increased annually. However, evidence regarding what is being carried out as rehabilitation treatment under LTCI is lacking. In this study, a scoping review was performed to bridge this knowledge gap.

**Methods:**

Articles related to rehabilitation in connection with LTCI published between April 2000 and November 2020 were searched for in PubMed, CINAHL, CENTRAL (Cochrane Central Register of Controlled Trials), Ichushi Web Ver.5, and CiNii and randomized controlled trials (RCTs) of rehabilitation provided under LTCI were examined.

**Results:**

Of the 15,572 publications identified, 15 RCTs, including rehabilitation treatment by physiatrists and therapists, met the eligibility criteria of our review and were included. The rehabilitation trials in the 15 RCTs varied and included balance training, exercise therapy, cognitive tasks, and activities such as singing and dancing. The results allowed us to focus on three categories: fall prevention, dementia, and theory and tools interventions related to occupational therapy practice.

**Conclusion:**

The focal points of attention in the rehabilitation treatment of LTCI were identified. However, the physical function, quality of life, and activities of daily living (ADL) of those who “need support” vary from person to person. Therefore, the consolidation of evidence on rehabilitation treatment of LTCI must be continued.

**Supplementary Information:**

The online version contains supplementary material available at 10.1186/s13561-022-00407-6.

## Background

In 2019, Japan’s population was 126.17 million, of which 35.89 million (28.4% of the total population) were between the ages of 65 and 75 years, and 18.49 million (14.7% of the total population) were over 75 years [1]. The proportion of older adults in the population is projected to increase as the number of people aged 65 and over increases, while the total population of Japan declines. By 2065, the percentage of the total population aged ≥ 65 and ≥ 75 years is estimated to reach 38.4% and 25.5%, respectively [1]. In the 1990s, the number of older adults requiring nursing care increased, and the shift to nuclear families changed the function of the family in nursing care. Caring for older adults became the greatest cause of anxiety in old age for both caregivers and those requiring care. In April 2000, the long-term care insurance (LTCI) act was enacted to prevent functional decline, enabling older adults to live independently in the community for as long as possible, and to expand community-based care [2–4].

The basic concepts defining LTCI comprise “self-reliance,” “user-oriented,” and “social insurance systems.” This “self-reliance support” includes rehabilitation as well as nursing care services that substantially support activities of daily living (ADL) and other aspects of life. Older individuals who wish to receive nursing care services, including rehabilitation, must be certified as requiring nursing care [5]. Initially, in the LTCI system, there were six levels of care requirements; however, after the LTCI reform in 2005, an additional level was added, with the lowest two levels classified as “needing support” and the remaining five levels referred to as “needing care” [6–8]. In the 15 years after the enactment of the LTCI act in 2000, the number of people certified as requiring long-term care almost tripled, from 2.18 million to 6.08 million, and the number of LTCI users is increasing annually [1–4].

Rehabilitation under LTCI is designed to provide older adults with the required support so that they can live as independently as possible. This rehabilitation for the purpose of “self-reliance support” includes rehabilitation treatment by specialists (therapists) under the supervision of rehabilitation physicians (physiatrists), rehabilitation by non-professional staff, and support by family members. Rehabilitation treatment under medical insurance incorporates the concept of disease-specific implementation as a part of treatment. Based on the available evidence, it is further developing in the direction of maintaining and improving the quality of life (QOL) for individuals with diseases and disabilities. In other words, rehabilitation medical care is essentially covered by medical insurance. Medical insurance is a system in which the insurer pays all or part of the medical expenses incurred by visiting a medical institution and is based on the National Health Insurance Act of 1958. However, the number of people requiring severe nursing care as well as older people with dementia, who also have medical needs, is increasing [1]. Hatano stated that the system linking medicine and long-term care is not functioning [9]. Considering rehabilitation from the perspective of those who use rehabilitation in the provision system of medical insurance and LTCI is necessary to seamlessly and efficiently provide rehabilitation that meets their needs. Furthermore, examining rehabilitation treatment in the LTCI field and accounting for future social changes are necessary, such as from the perspective of supporting independence, disease prevention, care prevention, and enabling people to live independently in their own homes [10–12]. However, the evidence on rehabilitation in the long-term care insurance domain is not organized.

This lack of evidence is partly because the LTCI act has not been in effect for a long time, as well as because the background of the target population varies considerably (e.g., homebound, with or without family members). Users often have multiple diseases, and there are various types of facilities, including some that focus on rehabilitation and others that provide only nursing-centered services. These factors are suspected to have led to an increase in the number of case studies examining individual subjects and a decrease in observational and experimental studies. The subjects of rehabilitation treatment for long-term care insurance are not treated to improve a single function, but rather the necessary exercise content is covered and implemented according to various symptoms to maintain and improve ADL. Therefore, the outcomes and treatment contents are not limited, but rather diverse, and rehabilitation treatment encompasses them all. A scoping review was determined to be an effective way to organize this content.

Prior research has reported the importance of distinguishing between reversible and irreversible multidimensional functioning of older adults at different levels of certification, to prevent future functional decline and provide efficient and effective care [13]. This study is a scoping review of studies on rehabilitation treatment conducted in the field of LTCI since the enactment of the LTCI act, allowing us to examine the elements necessary for rehabilitation treatment to support independence.

The results of this study may provide important insights not only for Japan but also for other countries. In the United States, as of 2015, more than 15,600 nursing homes reportedly provided care for older adults [14]. According to the National Bureau of Statistics of the People’s Republic of China, in 2016, the number of individuals aged 60 years and above reached 231 million, accounting for 16.7% of the country’s population [15]. The number of older individuals in need of nursing care is increasing both in Japan and abroad. This study may provide valuable information for countries with aging populations.

Therefore, a scoping review was conducted of studies related to rehabilitation treatment in the LTCI field reported in Japan to date. The study aimed to examine the initiative of rehabilitation treatment covered under the current LTCI scheme, by limiting the scope to experimental studies conducted in facilities that actively implement rehabilitation, and to clarify future issues by summarizing the year of the study report, age, and sex ratio of the subjects, as well as the study design, intervention programs, and their effects.

## Methods

### Eligibility criteria for study selection

Rehabilitation performed in the long-term care insurance domain can be divided into rehabilitation treatment and rehabilitation management. Rehabilitation therapy is carried out by therapists under the supervision of a physiatrist, whereas rehabilitation management is carried out by non-professionals working together. As the eligibility criteria for this study, the facility category that provides rehabilitation treatment was used. Day-care rehabilitation and visiting rehabilitation from hospitals, home visits, nursing homes, and rehabilitation (functional training) from special nursing homes are methods of providing rehabilitation in the long-term care insurance area (welfare facilities for older adults). The first three are staffed by physiatrists and therapists who provide rehabilitation treatment. Therefore, the inclusion criteria were studies related to rehabilitation covered by LTCI, with descriptions of evaluation, rehabilitation in long-term care facilities, day-care rehabilitation, and visiting rehabilitation from hospitals. The target clinical trials examined the effects of rehabilitation in the field of LTCI; the study design was a randomized controlled trial (RCT), and the articles were in the English and Japanese languages. Exclusion criteria were studies related to home visits, nursing homes, rehabilitation (functional training) from special nursing homes (welfare facilities for older adults), review articles, case reports, conference records on topics other than those concerning rehabilitation fields, and articles written in languages other than English and Japanese. In addition, publications related to dysphagia and swallowing training were excluded.

### Collection of research materials

The conduct of this scoping review was based on the framework and principles reported by Arksey and O’Malley [16], with additional recommendations provided by Levac, et al. [17]. This study was conducted using the following procedures: creation of a search formula based on the Preferred Reporting Items for Systematic Review and Meta-Analysis Extension for Scoping Reviews statement [18]; a reporting guideline for scoping reviews; and database search based on the search formula, primary screening, secondary screening, and analysis. The search formula was developed independently by five people, including the authors, doctors, physiotherapists, and occupational therapists. The primary screening of the title and abstract for all articles searched was carried out by two experts, and disagreements were judged separately by a third expert. The secondary screening involved examining the text and selecting articles by two experts, with disagreements resolved by a third expert.

Articles related to rehabilitation in the area of LTCI from April 2000 to November 2020 were searched for in PubMed, CINAHL, CENTRAL (Cochrane Central Register of Controlled Trials), Ichushi Web Ver.5, and CiNii. The search formula is presented in Online Resource 1. The selected articles’ bias risk was reviewed by two authors and approved by one. The co-authors also evaluated nonlinearity and publication bias.

## Results

**Fig. 1:**
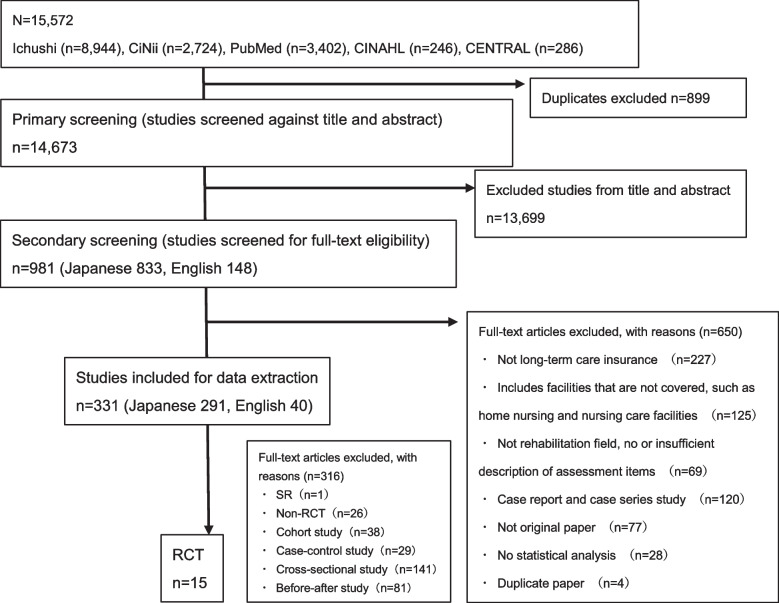
Preferred Reporting Items for Systematic Reviews and Meta-Analyses Extension for Scoping Reviews (PRISMA-ScR) flow chart

Figure 1 depicts the article search and the extracted results. After searching the databases, a total of 15,572 articles were extracted. This included 3,402 articles from MEDLINE (PubMed), 286 articles from CENTRAL, 246 articles from CINAHL, 8,944 articles from Ichushi Web, and 2,724 articles from CiNii. After primary screening, 981 articles were extracted. Thereafter, the full articles were obtained, and as a secondary screening, a full-text survey was conducted, and 331 articles were confirmed to be related to rehabilitation covered by LTCI. Of these, 15 articles with a research design of an RCT were selected for review (Online Resource 2). The 15 RCTs were broadly divided into three types: (1) rehabilitation provided for fall prevention [19–25], (2) rehabilitation provided for treating dementia [26–30], and (3) theories and tools related to the practice of occupational therapy [31–33].

When the 15 volumes considered were classified by reporting year, there were three volumes in 2005–2010, five volumes in 2011–2015, and seven volumes in 2016–2020. The average age of the subjects in the included articles was 66.5 years at the minimum and 84.4 years at the maximum. Regarding the sex ratio of the subjects, 1 volume was composed of 64.3% men [19], 11 volumes had a high percentage of women (52.4–90.0%) [26, 27], 2 targeted only women [20, 27], and 1 had an unknown subject sex composition [21]. As of October 2019, approximately 56.5% of Japan’s population was aged 65 and over, and 60.6% of the population aged 75 and over were women [1]. In addition, approximately 69.4% of the recipients of long-term care benefits were women [4]. Therefore, the age composition and the male-female ratio of the subjects in the articles collected in this literature survey were similar to the male-female composition of Japan as a whole.

### Fall prevention

The article with the shortest intervention period, which reported efficacy in improving balance ability, examined the effect of rehabilitation on trunk function for 4 weeks. They found that two trunk exercises, using machine training, performed two or three times a week at three sets of 10 repetitions each, for 4 weeks, resulted in significant improvement in the evaluation index of dynamic balance ability. However, no improvement was observed in the center of gravity sway in the standing posture [19]. In a study on foot care and foot grip strength training, 15 min of exercise twice a week for 12 weeks was suggested to improve foot flexibility, improve dynamic standing balance, and increase walking speed compared to foot grip strength training alone [20]. The longest training duration in a study was 6 months and consisted of an individual and group program combining several types of training [23], and a study combining elongation training, mainly self-training, and physical therapy for 10 min per session [24]. The latter had a high completion rate of 93%; it was as effective as physical therapy and effectively improved walking ability and balance. Three studies reported the effects of combinations of fall prevention interventions. Yamada et al. reported that an intervention that included balance training as well as cognitive tasks, such as calculation and reading sentences aloud, significantly reduced the incidence of falls [21]. In addition, a study that reported a combination of 12 exercise sessions plus a 3-month nutritional intervention also suggested that it was effective in preventing falls [22]. However, there was concern that in the group with reduced physical function, a greater number of exercise interventions may increase activity and, thus, result in falls. An intervention combining a multitarget stepping program with a diverse exercise program showed significant improvements in step accuracy, eye movement, and physical performance in a complex environment [25].

### Dementia

In the intervention involving reading aloud and singing, 16 participants in the reading-aloud group read short poems, 16 participants in the singing group sang nursery rhymes and songs, and 16 participants in the control group were each given a 20-min program twice a week for 3 months. As a result, the frontal assessment battery (FAB) scores, an evaluation index of frontal lobe function, were significantly higher in both the singing and reading-aloud groups. The mini-mental state examination (MMSE), a measure of cognitive function, and the FAB showed significantly higher scores after the intervention only in the singing group. The reading aloud program was expected to activate the frontal lobe, while the singing program was expected to improve cognitive and frontal lobe function [27]. In a study by Murai et al., 18 participants in the intervention group undertook a cooking program and 18 participants in the control group underwent a recreational program for 90 min once a week for 3 months [29]. The results showed that the Yamaguchi kanji-symbol substitution test, which is a measure of sustained attention and executive function, showed a significant decline in the control group but was maintained in the intervention group. The dementia behavior disturbance (DBD) scale, a measure of the behavioral and psychological symptoms of dementia (BPSD), showed a significant decrease in the intervention group. The DBD items of anxiety, agitation, and inhibition showed significant improvements in the intervention group. These results suggest that the cooking program intervention can reduce BPSD, maintain executive function, and positively affect mood [29]. In addition, as an intervention, Ishikawa et al. used dance therapy with pair dancing for a total of eight sessions that were 30–40 min each, conducted once a week. Resultantly, the MMSE scores of the intervention group increased post intervention, but no significant difference was observed between the intervention and control groups. In addition, no effect was observed on BPSD or ADL.

In general, electroencephalography of older people with dementia showed a slow-wave pattern with a decrease in the power of the alpha band and an increase in the level of the theta band. Further, salivary amylase activity increased after the first half of the dance therapy intervention but decreased after the second half. These findings suggest that dance therapy may be effective in improving cognitive, brain, and autonomic functions [28]. Tanaka et al. conducted a 12-week, twice-weekly intervention where 13 participants in the group intervention (GI) group received a 1-h intervention in a group session, 16 participants in the personal intervention (PI) group received a 20-min individual intervention, and 14 participants in the control group received usual care. For the intervention group, cognitive rehabilitation consisting of reminiscence therapy, reality orientation, and physical activity was conducted. A significant effect on the MMSE was observed in the GI group compared with the control group. An improvement trend in the Clinical Dementia Rating Scale–Sum of Boxes in the GI group was observed as well, which indicates the severity of dementia, while the PI group showed no effect [26]. In addition, 23 participants in the intervention group were given group recreational activities of “balloon volleyball, object relay, association game, and coloring book,” and 25 participants in the control group were given the intervention of “randomized programs, such as storytelling, quiz, and radio gymnastics by caregivers” for 30 min per session, twice a week [30]. As a result, significant interactions were found for “severity of BPSD” and “degree of burden for caregivers” in the neuropsychiatric inventory and “lively interaction with surroundings,” “expression of self,” and “control of difficult-to-respond behavior.” This suggests that group recreational intervention may improve or prevent BPSD progression, improve QOL, and reduce the burden on caregivers as BPSD improves [30].

### Theories and tool interventions in occupational therapy practice

A study on the efficacy of an occupational therapy program using occupational self-assessment (OSA)-II found the importance of goals and values in the report [32]. The study evaluated the outcomes of the goals set in OSA-II after 3 months of intervention; 34 subjects were equally divided into an experimental group and a control group for comparison. Consequently, several goals related to roles and daily routines were mentioned in the experimental group, and although the program focused on work, no difference was observed in the effects of the program between the experimental and control groups. However, some of the participants in the experimental group were closer to achieving their goals than others; those who were closer to achieving their goals tended to have higher health-related QOL. The RCT study using the management tool for daily life performance (MTDLP) was conducted with 114 subjects in the experimental group and 116 subjects in the control group, and no significant differences were observed in the ADL indicators in the experimental group [33].

Further, one study examined the MTDLP’s ADL application form [31]. The Barthel Index (BI) was significantly higher in the control group than in the intervention group, 83.37 ± 16.54 and 73.68 ± 19.90, respectively. The EuroQol Visual Analogue Scale (EQ-VAS) scores were significantly higher in the intervention group than in the control group, 65.57 ± 21.87 and 54.38 ± 20.59, respectively. No significant difference was observed between the two groups in terms of the Frenchay Activities Index (FAI) and The Tokyo Metropolitan Institute of Gerontology index of competence (TMIG-IC). No significant difference was observed in the time course of BI, FAI, TMIG-IC, and EQ-VAS scores before and at 3 months and 6 months post intervention.

## Discussion

### Fall intervention

According to the results of a survey conducted by the Ministry of Health, Labour and Welfare in 2021, falls and fractures accounted for 13.0% of the total causes of older adults needing nursing care, and it was the third largest cause after dementia and cerebrovascular disease [34]. Several older people experience falls in their daily lives. Fractures and intracranial hemorrhage triggered by falls lead to deterioration of living functions and death. Therefore, assessing the risk of falls in the older population and actively intervening to prevent falls in high-risk individuals are necessary. Indeed, the lower the level of care required, the higher the ADL level [8]. Those who are more active during the day and perform activities while sitting and standing have also been reported to have higher physical fitness [35]. Moreover, in nursing home residents, it is also true that as long as a person is active, there is a risk of falling, which cannot be avoided considering the age and physical strength of nursing home residents. Therefore, making a comprehensive judgment and continuing efforts to maintain and improve the living functions of residents without focusing solely on falls and fall-related injuries and deaths are necessary. The World Health Organization has developed guidelines for fall prevention measures [36]. In addition, the evidence on fall prevention in community-dwelling older adults is increasing [37–40].

There were seven RCT studies on fall prevention under LTCI. This may indicate growing interest in fall prevention. RCT studies on fall prevention under LTCI included training related to physical functions such as balance ability, trunk muscle strength, and toe muscle strength, as well as eye movement, with some even including a multitarget stepping program. A complex program that includes elements other than physical function may be a necessary component of fall prevention. In addition, the frequency of training should be at least twice a week for 1 month, with a long-term intervention period of 3 or 6 months. Other studies involving with older adults’ reports with older adults’ reports include studies in which training was conducted for 12 and 24 months, and the effects of long-term implementation are high [41, 42]. Although effective training periods cannot be examined from the results of this study, longer-term training programs may be considered. It is also interesting to note that we found an initiative on toe exercises under LTCI. In Japan today, even for older people who can be expected to benefit from exercise, the intervention is limited to the use of day-care rehearsals and does not extend to the provision of guidance on self-directed training at home because of a few risks such as falling. Training that can be performed safely without supervision, such as toe exercises and foot care, may be a solution [43, 44]. The contents of the program must be examined so that the program can be performed safely at home.

The fall prevention studies selected did not report effect sizes, and the clinical significance of the intervention effect is unclear. Additionally, only one article also presented 95% confidence intervals [25]. Hence, future studies should report effect sizes and 95% confidence intervals.

### Interventions for dementia

The LTCI act introduced the measure of “intensive rehabilitation for newly admitted persons with dementia” in 2006 [45]. In January 2015, the government created the Comprehensive Strategy for the Promotion of Dementia Policies (New Orange Plan), which aimed to promote knowledge and awareness of dementia, provide timely and appropriate medical and nursing care, and create older adult-friendly communities, with the goal of enabling older people with dementia to live in their own communities [4]. The effects of physical activity in rehabilitation for dementia have been reported in several studies. Reportedly, regular physical activity is associated with a lower incidence of dementia and Alzheimer’s disease [46, 47], and physical activity intervention trials in older people without dementia and with mild cognitive impairment reduced cognitive decline [48–50]. These studies report that leisure activities (games, movies, etc.) and social participation (meeting friends, volunteering, etc.) are effective as well [51–53].

There were five RCT studies on dementia under LTCI. These included a wide variety of trainings, rather than one common training. As described above, most RCT studies on LTCI-covered interventions for rehabilitating patients with dementia reported improvements in cognitive function, followed by improvements in BPSD. Interventions that use a variety of recreational activities, such as singing and dancing, have the potential to increase enjoyment and pleasure. The perspective of enjoyment and pleasure, such as the inclusion of songs for group exercises while having fun, is assumed to be a factor that supports its continuation. Reports indicated that health promotion programs including facial expression muscle training improved QOL in LTCI, which may provide evidence for expressing enjoyment and pleasure [54]. Of the five reports selected for this study, only two articles [27, 28] reported effect sizes, and the extent to which the intervention effects are clinically significant remains unclear. In the future, the effect sizes and 95% confidence intervals must be reported.

### Theories and tool interventions related to occupational therapy practice

Since the 1980s, the advancement of occupational science has led to the development of many theories and tools related to occupational therapy. Typical theoretical models of occupational therapy that are well known in Japan include the Canadian model of occupational performance developed by the Canadian Association of Occupational Therapists [55] and the model of human occupation [56]. In addition, the occupational therapy intervention process model [57] shows the occupational therapy intervention process and framework. Tools include the Canadian occupational performance measure [58] and the model of human occupation screening tool [59]. Some of these tools related to occupational therapy practice were developed based on specific occupational therapy theories, while others were developed without a specific foundational theory. The Japanese Association of Occupational Therapists developed the MTDLP, which is a comprehensive occupational therapy practice tool that presents a framework clarifying the practical process of occupational therapy and provides various sheets that enable practice according to the framework.

The three selected papers have one thing in common: they all used goal-oriented sheets to improve quality of life and ADLs. One study discovered no difference in health-related quality of life or ADL [32]. OSA II, a self-assessment support tool based on the Model of Human Occupation (MOHO), is the main intervention method used by Ishidai et al. [32]. This includes self-assessment questions about “how well I am doing” in determining goals and accomplishments. In this regard, the patient’s inability to increase the activity level is believed to be due to the influence of cognitive function and emotions. By contrast, Omori et al. [31] and Noto et al. [33] used MTDLP in their study and implemented a tailored rehabilitation program; they discovered that both health-related quality of life and ADL were improved. When using the MTDLP for evaluation, a specific treatment plan was developed for each of the four levels: fundamental practice, basic practice, applied practice, and social adaptation practice, according to a sheet called the “Activity Improvement Plan.” In addition to some staged training programs, guiding them may be necessary. The findings of these two studies indicate that the use of MTDLP is associated with improved ADLs. However, the fact that the subjects were older adults with higher BI scores and lower levels of care prior to the intervention could have influenced the results.

From these viewpoints, theories and tool interventions related to occupational therapy practice are incorporated in LTCI. However, their effectiveness under LTCI needs to be examined in the future.

## Limitations

The limitations of this study are as follows. First, this study aimed to provide evidence to guide rehabilitation interventions covered under LTCI for individuals whose level of independence is expected to improve; thus, the study was limited to only those facilities that actively provide rehabilitation programs covered by LTCI. Therefore, the efficacy of rehabilitation for individuals with more severe conditions, such as bedridden individuals, was not covered. Second, as LTCI is a rare system worldwide, it may not be compatible with other countries. However, older individuals worldwide require nursing care, and many need rehabilitation to maintain or improve their independence.

Third, this study was not able to examine the issue from the perspective of collaboration among professionals related to rehabilitation. Physical, occupational, and speech therapists are involved in some rehabilitation programs covered by LTCI. Mutual collaboration (liaison) among professionals has been reported to be highly effective in in-home rehabilitation, but this factor was not examined [60]. Ideally, interventions should be defined and reviewed based on the mechanism of intervention. However, the interventions in rehabilitation medicine vary widely, and determining which treatment is more effective is difficult from this study.

## Conclusion

This scoping review was conducted to examine the available evidence to guide future rehabilitation treatment for persons with low levels of care provided under LTCI. As a result, fall prevention, dementia, ADL, and QOL were determined to be important factors.

In this study, the rehabilitation conducted under LTCI was analyzed from three perspectives: “prevention of falls,” “dementia,” and “theories and tool interventions.” Fall prevention was discussed in several papers among those selected. Intervention methods for dementia vary, and presented tasks should include an element of enjoyment. This study also identified interventions with theories and tools related to occupational therapy practice. However, the examination of evaluation sheets and other tasks needs to be continued. In the future, a systematic review of the three points categorized in this study must be conducted. Furthermore, the examination of the concept of rehabilitation treatment under long-term care insurance, which was carried out in this study, must be continued.

## Supplementary Information

**Additional file 1.**

**Additional file 2.**

## Data Availability

All data generated or analyzed during this study are included in this published article and its supplementary information file.

## Abbreviations

LTCI: Long-term care insurance
RCT: Randomized controlled trial
ADL: Activities of daily living
QOL: Quality of life
CENTRAL: Cochrane Central Register of Controlled Trials
FAB: Frontal assessment battery
MMSE: Mini-mental state examination
DBD: Dementia behavior disturbance
BPSD: Behavioral and psychological symptoms of dementia
GI: Group intervention
OSA: Occupational self-assessment
MTDLP: Management tool for daily life performance
