# Supplementary material for: Rehabilitation in the long-term care insurance domain: a scoping review
Source: Health Econ Rev. 2022 Dec 1;12:59. doi: 10.1186/s13561-022-00407-6 (PMC9713971; doi:10.1186/s13561-022-00407-6)
Supplement: Supplementary file 1 — Additional file 1. [file 13561_2022_407_MOESM1_ESM.docx]

**Rehabilitation in the Long-term Care Insurance Domain: A Scoping Review**

|  | Search words |
| --- | --- |
| P | “insurance, health” OR “insurance, long term care” OR (“day care, medical” OR “Ambulatory Care Facilities”) OR (“house calls”) OR (“Residential Facilities”[MeSH]) OR “cerebrovascular disorders” OR “heart diseases” OR (“bone and bones” OR “joint diseases”) OR “dementia” OR “fractures, bone” OR “accidental falls” OR “pneumonia, aspiration” OR (“Disuse syndrome” OR “muscular disorders, atrophic” OR “Sedentary Behavior” OR “Cardiovascular Deconditioning” OR “Immobilization”) OR “community based rehabilitation” OR (“Health Maintenance Organizations” AND “rehabilitation”) OR (“convalescence” AND “rehabilitation”) OR (“Disease-specific” AND “rehabilitation”) OR ((“acute” OR “acutely” OR “acutes”) AND “rehabilitation”) OR “aphasia” OR (“executive function” AND “disease”) OR “deglutition disorders” OR “pulmonary disease, chronic obstructive” OR “Aged” |
| I | “Therapy” OR “Management tool daily life performance” OR “independent living” OR “social participation” OR “leisure activities” OR “hobbies” OR “Community Participation” OR “Cognitive Behavioral Therapy” OR (“executive function” AND “rehabilitation”) OR “employment, supported” OR “equipment and supplies/supply and distribution” OR “deglutition disorders/rehabilitation” OR “self help devices“ |
| O | “muscle strength” OR “walking speed” OR “timed up and go test” OR “Short Physical Performance Battery” OR “sit to stand test” OR “range of motion, articular” OR “Walk Test” OR “Barthel index” OR “Functional Independence Measure” OR “lawton instrumental activities of daily living” OR “Frenchay Activities Index” OR “world health organization disability assessment schedule 2 0” OR “repetitive saliva swallowing test” OR “modified water swallow test” OR “speech intelligibility” OR “dependency, psychological” OR “care level” OR “Zarit Burden Interview” OR “Quality of life” OR “fee for service plans” OR “long term care/economics” OR “mini mental state examination” OR “hasegawa dementia scale revised” OR “kohs block design test” OR “Trail making test” OR “Vitality Index” OR “Self-rating Depression Scale” OR “Hospital Anxiety and Depression scale” OR “frontal assessment battery” OR “Dementia Assessment Sheet for Community-based Integrated Care System-21 items” or “Dementia Behavior Disturbance Scale” or “Standard Language Test of Aphasia” |

**Online Resource 1**

Search for “P,” “I,” and “O” with “AND”
