# Supplementary material for: Rehabilitation in the long-term care insurance domain: a scoping review
Source: Health Econ Rev. 2022 Dec 1;12:59. doi: 10.1186/s13561-022-00407-6 (PMC9713971; doi:10.1186/s13561-022-00407-6)
Supplement: Supplementary file 2 — Additional file 2. [file 13561_2022_407_MOESM2_ESM.docx]

**Rehabilitation in the Long-term Care Insurance Domain: A Scoping Review**

**Online Resource 2**: Studies included in this review

| Author (year) | Target facility | Subjects (experimental group) | Subjects（control group） | Average age | Intervention period | Exercise program | Evaluation index | Outcome | Abbreviations |
| --- | --- | --- | --- | --- | --- | --- | --- | --- | --- |
| Yamada et al. (2008) | Long-term care health facility | DT group: 22 subjects ST group: 21 subjects | 22 subjects | 84.4±5.4 years | Unknown | DT group: Balance training: 10 min, 2 times/week, 12 weeks + cognitive tasks (calculation task and reading aloud task) ST group: balance training: 10 min, 2 times/week, 12 weeks | Physical function assessment: TUG, single leg standing time, FRT, 10mWT, 10mWT under DT conditions, Δ10mWT | ST group: TUG↑, single leg standing time↑,FRT↑, 10mWT↑, 10mWT under DT condition↑(p<0.05) DT group: TUG↑, single leg standing time↑, FRT↑, 10mWT↑, DT condition 10mWT↑,Δ10mWT (p<0.05) Control group: → | DT: Dual-task ST: Single-task  FRT: functional reach test 10mWT: 10 m Walking test TUG: Timed Up & Go test |
| Imaoka et al. (2015) | Long-term care health facility | Exercise intervention group: 23 subjects (82.5±10.9) Nutritional intervention group: 23 subjects (87.6±6.5) | 22 subjects (82.6±9.1) | 84.3±9.2 years | Oct. 1, 2013-Dec. 31, 2013 | Exercise intervention group: Usual institutional care + exercise therapy in small groups of 10 or fewer people (30 min, once/week, total of 12 levels) Control group: Usual institutional care Nutritional intervention group: Usual institutional care + vitamin D intake of at least 800 IU/day | SMI, grip strength, 25(OH)D, FIM | Nutrition intervention group: 25(OH)D ↑, Other items→ Incidence of falls: control group: five subjects (22.7%), exercise intervention group: eight subjects (47.1%), nutrition intervention group: two subjects (10.0%) | SMI: Skeletal Muscle Mass Index 25(OH)D: 25 hydroxyvitamin D  FIM: Functional Independence Measure |
| Yamada et al. (2013) | Long-term care health facility | 132 subjects (76.2±8.5) | 132 subjects (77.2±7.6) | Unknown | Unknown | Community-based MTS program + standardized multi-component exercise program (Twice a week, 24 weeks) | Number of falls, number of fractures related to falls, step accuracy, eye movement during MTS test, TUG, FRT, 10mWT, 5CS | Falls: 13 (11.6%) in the MTS group and 39 (33.0%) in the control group (incidence ratio 0.35, 95% confidence interval [CI] 0.19-0.66) Fractures related to falls: 3 in MTS group, 13 in control group (relative risk 0.22, 95%a = 0.06-0.80) MTS group: accuracy of step during test↑, eye movement↑, TUG↑, 10mWT↑ (P < 0.001) | MTS: Multitarget stepping TUG: Timed Up and Go test FRT: Functional Reach Test 10mWT: 10 m Walking test 5CS: 5 chair stands |
| Takahashi et al.（2017） | Day-care rehabilitation service | 13 subjects (86.5±4.9) | 12 subjects (80.3±8.0) | 83.5±6.5 years | Unknown | ELT group: 10 min of ELT + 10 min of physical therapy excluding ROM and muscle strengthening ex. twice/week, 6 months Control group: ROM practice, muscle strengthening practice, standing movement practice, walking practice, etc. | Height, weight, grip strength, Quadriceps femoris strength, CS-30, active hip extension angle, active shoulder flexion angle, Sit&Reach test, single leg stand time, TUG, MWS , 2ST | Both groups: quadriceps femoris strength↑, CS-30↑, active hip extension angle↑, Sit&Reach↑, TUG↑, 2ST↑. | ELT: Elongation training CS-30: 30-sec chair-stand TUG: Timed Up and Go test  MWS: maximum walking speed  2ST: 2 Step Test |
| Shimanuki et al. (2007) | Long-term care health facility | 7 subjects | 7 subjects | 70.8±8.4 years | Unknown | PR group: usual physical therapy program + power rehearsal for trunk Control group: usual physical therapy only | Muscular Strength of the Trunk, Measurement of center of gravity motions, FRT, TUG, 10 m walking speed | PR group：muscular strength of the trunk↑, FRT↑ Group C: All items → | PR: Power rehabilitation FRT: Functional Reach Test TUG: Timed Up & Go Test |
| Yasuda et al. (2014) | Day-care rehabilitation service | Foot care and grasping strength training group: 18 subjects (83.4±5.4) Foot grasping strength training group: 15 subjects (83.1±6.7) | 16 subjects (83.65±6.7) | 84.0±6.2 years | Unknown | 12 weeks, 15 min, 2 times/week foot bath + 10 min of stretching and massage, 5 times of foot grip strength training, 2 times/week | Foot grip strength, foot flexibility, foot length, FRT, center of gravity motions, walking speed, grip strength, Quadriceps femoris strength | Combined foot care and training group: foot grip strength↑, foot flexibility↑, FRT↑, walking speed↑ (VS training group, control group) Training only group: foot grip strength↑, FRT↑, walking speed↑ (VS control group) Control group: all items → (VS control group) | FRT: Functional Reach Test |
| Sugiura et al. (2010) | Long-term care health facility | 10 subjects (78.9±5.8) | Control group that did only individual exercise program: 10 subjects (80.0±8.6) | 79.5±7.2 years | Unknown | Intervention group: Individual exercise program [5 out of 13 individual training items (8 strength training items and 5 stretching items) (but 2 stretching items), 3 sets of 10 times each, 5 times/week] and group rhythm exercise Control group: Individual exercise program only | Grip Strength, Maximum Isometric Knee Extension Strength, Sit&Reach test, Eye Opening single leg Stand Time, FRT, TUG, Comfortable and Maximum Walking Speed, Falls Efficacy | Both groups: Strength of lower limb musclesup Intervention group: Balance ability↑, Walking ability↑, Mental function | FRT: Functional Reach Test TUG: Timed Up & Go Test |
| Ishikawa et al. (2014) | Long-term care health facility | 9 subjects (82.4±7.5) | 9 elderly patients with dementia (77.6±11.6) | Unknown | November-December 2013 | Pair dancing to music for 30 min per session, once a week for a total of 8 sessions. | Cognitive function (MMSE, verbal fluency task, PAS), BPSD and ADL (NPI, N-ADL), sNAT, SAA | Cognitive function → (VS control group) (large effect size of MMSE in experimental group) BPSD and ADL→ (VS control group) EEG (sNAT) improved by intervention Salivary amylase by intervention (first half session ↑, second half session ↓) | BPSD: Behavioral psychological symptoms of dementia N-ADL: N-style assessment scale for the ability to perform activities of daily living for the elderly PAS: Psychogeriatric Assessment Scales NPI: Neuropsychiatric Inventory sNAT: electroencephalogram SAA: salivary amylase activity level |
| Honda et al.（2018） | Long-term care health facility | Singing group: 16 subjects (87.1±5.2) Reading aloud group: 16 subjects (86.9±4.6) | 11 subjects (88.5±5.0) | Unknown | Unknown | Reading aloud or singing program of 20 min per session, twice a week for 3 months (total of 24 sessions, 480 min) | MMSE, FAB, NM scale, N-ADL, STAI | Reading aloud group: MMSE→, FAB↑, NM scale→, N-ADL→, STAI→. Singing group: MMSE↑, FAB↑, NM scale→, N-ADL→, STAI→. Control group: No change before and after No significant difference between the three groups | MMSE: Mini Mental State Examination FAB: Frontal Assessment Battery STAI: State-Trait Anxiety Inventory NMスケール：N-style mental state scale for the elderly N-ADL: N-style assessment scale for the ability to perform activities of daily living for the elderly STAI: State Trait Anxiety Inventory Japanese version |
| Sakamoto et al. (2017) | Long-term care health facility | 17 elderly people with dementia (85.6±6.3) | 12 elderly people with dementia (87.9±6.2) | Unknown | Unknown | One 30-min group recreation intervention (Balloon volleyball, object relay, association game, pasting picture) twice a week for three months | MMSE、NPI、QOL-D | There is an interaction between NPI “severity of BPSD” and “level of burden for caregivers” and QOL-D “lively interaction with surroundings,” “expression of self,” and “control of difficult-to-respond behavior.” | MMSE：Mini Mental State Examination NPI: Neuropsychiatric Inventory QOL-D:QOL scale for the elderly with dementia |
| Murai et al. (2017) | Long-term care health facility | 18 elderly people with dementia (86.1±5.8) | 18 subjects (84.7±7.2) | Unknown | June-September 2011 | One 90-min cooking program intervention, three times a week, for three months | Word fluency, YKSST, RBANSstory, GDS, DBD (items 10, 22, 23, 24, 27), J-ZBI, PGC, Lubben, BI | YKSST: intervention group →, control group ↓ DBD total: intervention group↑, control group→ Item 10: intervention group↑, control group→ Item 22: Intervention group→, Control group↓ Item 23: Intervention group↑, Control group→ Item 24: Intervention group↑, Control group→ Item 27: Intervention group↑, control group→ No significant interaction for other items | YKSST: Yamaguchi kanji-symbol substitution test RBANS: Japanese version of the Repeat- able Battery for Assessment of Neuropsychological Status  DBD: dementia behavior disturbance GDS: 15-item geriatric de- pression scale J-ZBI: Japanese version of the Zarit caregiver burden interview PGC: Philadelphia geriatric center morale scale BI: Barthel Index Lubben: Lubben social network scale |
| Tanaka et al. (2017) | Long-term care health facility | GI group: 13 subjects (84.9±6.6) PI group: 16 subjects (86.0±7.4) | 14 subjects in the control group receiving usual care (86.5±8.3) | Unknown | Unknown | Cognitive rehabilitation consisting of reminiscence therapy, reality orientation, and physical activity; GI group (1 h per session, twice a week for 12 weeks) PI group (20 min per session, twice a week for 12 weeks) | CDR-SB、NOSGERtotal、QOL-D、MMSE、GDS-5、BCAS | MMSE: GI group↑, PI group→ CDR-SB: GI group↑, PI group→. No change in NOSGER, VI, QOL-D, GDS-5, and BCAS in both groups. | CDR-SB: sum of boxes in CDR NOSGERtotal: Nurses’ Observation Scale for Geriatric Patients QOL-D: health-related quality of life questionnaire for the elderly with dementia in Japan MMSE：Mini Mental State Examination GDS-5: Geriatric Depression Scale 5-item version BCAS: brief communication ability scale GI: Group intervention PI: Personal intervention |
| Noto et al. (2014) | Day-care rehabilitation | 114 subjects (75.8±9.9） | 111 subjects (77.9±8.9） | Unknown | September to December, 2011 | Rehearsal with management for improvement of activities of daily living, 1.5/W, 25min, 3M Usual OT, 1.5/W, 23.5min, 3M | BI, FAI, HUI | Experimental group: BI↑, FAI↑, HUI↑ Control group: BI→, FAI↑, HUI→ | BI: Barthel Index FAI: Frenchay Activities Index HUI: Health Utilities Index |
| Ishidai et al. (2016) | Day-care rehabilitation | 17 subjects (67.4±11.0) | 17 subjects (65.5±12.2) | 66.5±11.5 years | Unknown | Rehabilitation using OSA II, 9/M, 3M Individual Occupational Therapy,8/M, 3M | SF-36, QOL26, FIM, FAI | No change in all items in both experimental and control groups | SF-36: MOS 36-Item Short-Form Health Survey  QOL26: WHO QOL26 FIM: Functional Independence Measure FAI: Frenchay Activities Index |
| Omori et al.（2018） | Day-care rehabilitation | 31 subjects（81.97±8.49） | 32 subjects（81.59±11.72） | Unknown | August-November 2014 | Experimental group: occupational therapy based on management for improvement of activities of daily living, Control group: individual rehabilitation, group rehabilitation | BI, FAI, The Tokyo Metropolitan Institute of Gerontology index of competence, EQ-5D-5L, EQ-VAS, Execution of life goals, Satisfaction with life goals | Experimental group: BI↑, degree of implementation of life goals and satisfaction after 3 and 6 months↑. | BI: Barthel Index FAI: Frenchay Activities Index The Tokyo Metropolitan Institute of Gerontology index of competence: TMIG-IC EQ-5D-5L: EuroQol 5 dimensions 5-level EQ-VAS: EuroQol Visual Analogue Scale |
